# Modified A-Star Algorithm for Efficient Coverage Path Planning in Tetris Inspired Self-Reconfigurable Robot with Integrated Laser Sensor

**DOI:** 10.3390/s18082585

**Published:** 2018-08-07

**Authors:** Anh Vu Le, Veerajagadheswar Prabakaran, Vinu Sivanantham, Rajesh Elara Mohan

**Affiliations:** 1 Engineering Product Development, Singapore University of Technology and Design, Singapore 487372, Singapore; leanhvu@tdt.edu.vn(A.V.L.); prabakaran@sutd.edu.sg(V.P.); vnu.619@gmail.com(V.S.); 2 Optoelectronics Research Group, Faculty of Electrical and Electronics Engineering, Ton Duc Thang University, Ho Chi Minh City 7000000, Vietnam

**Keywords:** reconfigurable mechanism, floor cleaning robot, area coverage, path planning, feature mapping

## Abstract

Advancing an efficient coverage path planning in robots set up for application such as cleaning, painting and mining are becoming more crucial. Such drive in the coverage path planning field proposes numerous techniques over the past few decades. However, the proposed approaches were only applied and tested with a fixed morphological robot in which the coverage performance was significantly degraded in a complex environment. To this end, an A-star based zigzag global planner for a novel self-reconfigurable Tetris inspired cleaning robot (hTetro) presented in this paper. Unlike the traditional A-star algorithm, the presented approach can generate waypoints in order to cover the narrow spaces while assuming appropriate morphology of the hTtero robot with the objective of maximizing the coverage area. We validated the efficiency of the proposed planning approach in the Robot Operation System (ROS) Based simulated environment and tested with the hTetro robot in real-time under the controlled scenarios. Our experiments demonstrate the efficiency of the proposed coverage path planning approach resulting in superior area coverage performance in all considered experimental scenarios.

## 1. Introduction

Robots are fast becoming an integral component of our everyday life and being deployed towards a number of processes over a wide range of applications. Such robots are required to find safe and feasible routes to navigate effectively in the environment. This need is particularly crucial when these robots are navigating in complex and uncertain settings. In order to achieve efficient navigation, a robot must be equipped with necessary control units, sensor systems and the effective coverage path planning intelligence. Path planning is a method that regulates the robot’s path that passes over all parts of an area while avoiding obstacles. Path planning becomes an essential ingredient for many robotic applications, such as cleaning, painting, inspection and mining in order to amplify their performances. Over last few decades, different path planning methods have been proposed and demonstrated on robots with various applications. One of the most frequently used path planning methods is cellular decomposition which breaks the obstacle-free places into a non-overlapping region. These regions are called cells which is easy to cover and require simple motions (for instance Zigzag) to sweep the whole area. In literature [1,2,3], the authors documented the generation of simple motions using the cellular decomposition method. A simple decomposition method was proposed by Timo Oksanen and Arto Visala [4]—called the trapezoidal decomposition—to solve the path planning problem in agricultural machines. In this work, they used a top-down approach and split the complex agricultural fields into simple regions (cells) to navigate with elementary motions in order to cover the filed effectively. In Reference [5], Howie Choset and Philippe Pignon proposed a new approach called boustrophedon cellular decomposition which works similarly to other decomposition methods. However, the proposed method reduced the number of cellular cells compared to trapezoidal algorithm hence, the robot’s shorter coverage path is obtained. Ercan U. Acar presented a novel cellular decomposition method in Reference [6], wherein they used Morse function in order to indicate the location of the cell boundaries. In this study, the authors change the Morse function that modifies the path pattern of the robot to cover its free space. As an extension of this study, authors also presented a sensor-based coverage algorithm that uses cellular decomposition in terms of critical points of Morse function in Reference [7]. In this work, they identify the features of the proposed algorithm to eliminate the error sensor data in unstructured environments beyond performing sensor data processing. Galceran and Carreras presented a coverage path plan method based on the Morse decomposition function for an underwater surface robot [8]. This work aims to determine the best sweep orientation for each separated cell and the inter-lap spacing in the generated path on a lap by lap basis with respect to the ocean depth. Also, they validated the proposed algorithm in simulation experiments with real-time bathymetric data sheet. Similarly, H. Choset et al. [9] implemented a novel Morse cellular decomposition function combined with GVD (General Voronoi Diagram) algorithm in a space inspection robot. The presented work introduces a method to generate robot’s path in the three-dimensional space that reduces its fuel cost to navigate from one point to another. An alternative algorithm for the Morse decomposition method was proposed in Reference [10]. In this work, the author presents a landmark-based topological coverage in which the natural landmarks are added as nodes in the mapped space. In addition, they benchmarked their proposed path planning approach with the Morse based method and demonstrated higher coverage performance with the proposed scheme.

Utilization of grid-based methods for coverage path planning opens a lot of opportunity for research and development in the field of robot navigation. There are numerous such algorithms were proposed for efficient path plan for mobile robots. For Instance, Reference [11] presented a novel grid-based coverage approach where they considered time and energy as critical parameters to reduce directional constraints on path generation. The proposed approach was validated by benchmarking its performance to a conventional coverage scheme with respect to energy and time. In another grid-based coverage work, Joon Seop Oh et al. [12] proposed a novel grid pattern where they used triangular cells instead of rectangular cells for efficient and faster navigation in cleaning robots. The efficiency of the proposed algorithm was validated through simulated experiments. Coverage task with multi-robot scenarios have a higher advantage by utilizing coverage path planning algorithm. For multi-robot coverage, Pooyan Fazli et al. proposed multi-robot area coverage approach for a scenario in which a map is known with the minimum visibility range [13]. In this work, author initially locates the beacons in a known map to create a graph then they convert the generated graph into a forest of partial spanning tree (PST). The converted PST is then built as cycles which is then assigned to each robot for coverage. In an another multi-robot coverage work, Chaomin Luo and S.X. Yang proposed a bio-inspired neural network approach for robots operating in a time-varying and unstructured environment [14]. The prosed model will generate the shunting neural equation which provides the path for each robot through dynamic activity landscape for efficient coverage. Author claims the proposed method is computationally efficient than traditional methods. Although numerous methods in coverage path planning have been proposed and demonstrates its significant in area coverage task, they are largely tested with fixed morphological robots. None of the previous works in coverage path planning was applied or proposed specifically for robots with shapeshifting capability.

The reconfigurable mechanism is well studied and were actively applied to robotics platforms since 1980. Such efforts later translated to a number of reconfigurable robotic platforms that has been proposed. So far in the field of reconfigurable robots, three different architectures were proposed, namely, intra-reconfiguration, inter-reconfiguration and nested reconfiguration. Intra-reconfiguration deals with a single robotic system that could change its morphology by its own without any external supports. For instance, a versatile robot that could change its morphology from amphibious and terrestrial gait mechanism [15], a reconfigurable Janson mechanic robot that could generate a variety of gait patterns [16] and a bio-inspired crawling, rolling, climbing reconfigurable robot. Robots that was proposed under the inter-reconfigurable principle are basically modular robots that could possess different morphologies by undergoing assembling and disassembling process. One such example is Sambot [17], which can assume morphologies by attaching and detaching with multiple similar robots. CEBOT, Poly Bot, Crystalline, M-TRAN, ATRON, Molecube and CKBot are other relevant examples of inter-reconfigurable robots. The third architecture category in reconfigurable systems is Nest reconfiguration which is capable of performing both inter and intra reconfigurations. Hinged Tetro present in Reference [18], is the only such nested reconfigurable robot which is capable of switching between forms and could change its morphology by undergoing assembling and disassembling with peer robots. In spite of the fact that numerous studies in the literature address reconfigurable robotics, they are primarily limited to mechanism design and were never implemented to an area coverage task like floor cleaning. Also, none of the previous work in reconfigurable robot was validated with coverage path planning technique.

To this end, in our previous work, we presented a novel reconfigurable floor cleaning robot called hTetro that can able to change its morphology to any of the one-sided Tetris pieces [19,20]. The developed hTetro robot applies polyominoes tiling theory [21] as an autonomous coverage path planning strategy. The polyominoes tiling theory deals with the problem of partitioning or filling of a geometrical region using same or multiple polyominoes pieces under a particular case. The hTetro platform can automatically generate a global tiling set required to cover a defined space while leveraging on the polyominoes tiling theory. In that work, we validated the hTetro robot with respect to area coverage by benchmarking its performance with a fixed morphology robot. The results indicated that hTetro robot could achieve superior coverage performance through its shapeshifting ability. However, the validation was done by passing manual commands through an android app and there were no autonomous strategies applied. The main contributions of this paper are threefold. First, we extend our previous works by integrating the onboard LiDAR sensing modules and manipulations modules with the Tetris inspired hTetro on ROS environment that enables the robot to generate the path and shape plan to navigate autonomously. Second, the proposed method uses characteristics of the map built by ROS to find the different types of waypoints including boundary waypoints, obstacle waypoints autonomously. Then the A-star [22] based zigzag scanning pattern connects waypoints to cover the maximum free space areas and avoid the obstacle autonomously. Third, we demonstrated the transformation ability of hTetro between by switching back and forth I and O shapes to cover the auto-detected narrow areas such as spaces under tables, chair, corner, which is one of the significant challenges among the fixed morphology robots. The presented planning technique could automatically generate waypoints in order to cover the narrow spaces while assuming appropriate morphology of the hTtero robot with the objective of maximizing the coverage area. This paper includes an outline of the hTetro robot’s architecture design, onboard Simultaneous Localization and Mapping (SLAM) system and the challenges that encountered during the translation of theoretical design to realization of the proposed technique in real time. Moreover, this paper also summarizes the experimental setup to validate the proposed method with hTetro robot and concluded with the results that show the superior area coverage performance of the same.

## 2. On-Board-LIDAR-Sensor hTetro Hardware Architecture Configuration

**Figure 1:**
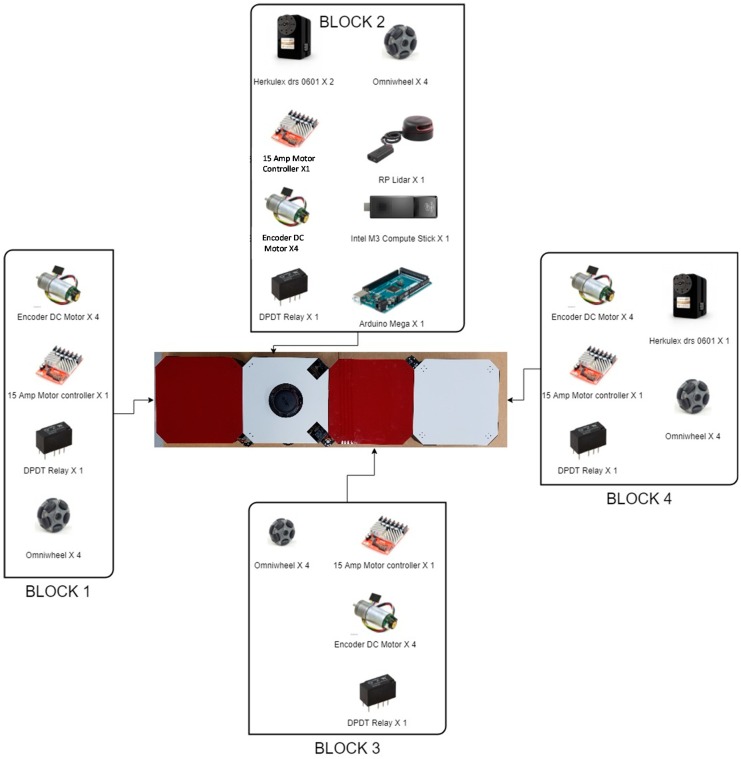
hTetro 5.6_Components List.

**Figure 2:**
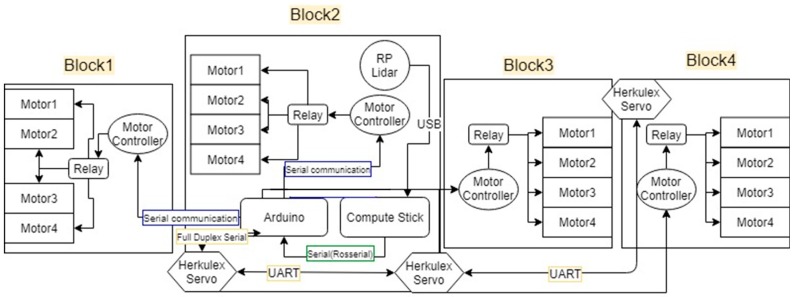
hTetro Hardware Architecture.

**Figure 3:**
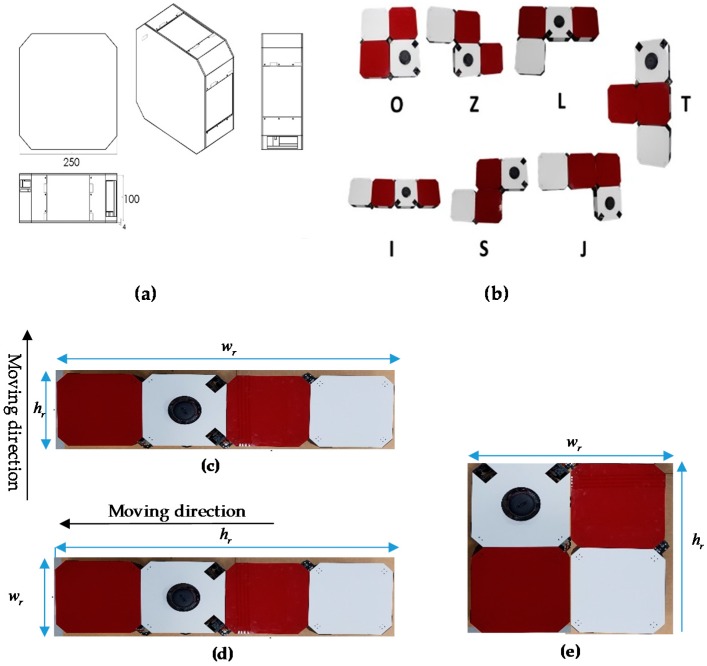
hTetro Single Block Dimension and morphologies. (**a**) Single Block Dimension, (**b**) seven hTetro morphologies, (**c**) I shape in vertical moving direction, (**d**) I shape in horizontal moving direction, (**e**) O shape.

The Figure 1 describes the hardware parts of the hTetro robot. The hinges between each block hold up together and are responsible for shape transformation during reconfiguration. The perception component of hTetro is one rpLidar mounted on block 2. The connections of hTetro hardware architecture are described in Figure 2. Making the robot stable during the locomotion was one of the primary objectives while designing hTetro robot. In detail, for more stable and balanced locomotion each box is mounted with four geared dc motors. A strong acrylic sheet of 4 mm thickness is used as the base of each block to allocate all the dc motors with other peripheral devices. The dc motor used in the hTetro for locomotion operates with a voltage rating of 7.4 V. Each of the 16 dc motors are programmed to work differently based on the transformation of the robot. When it comes to the transformation of the robot the smart servos mounted to the hinges that drive the blocks linked to the servo motors. The servo motors require a voltage of about 14.8 volt in order to perform effectively. Each servo motor has a stall torque of 77 kg.cm which is enough to drive the blocks during transformation and lock the position of the blocks after the self-reconfiguration. Two of the three servo motors are placed in block 2 and the remaining one set in block 4 connects block 3. The dimensions of all the four boxes are the same as given in the Figure 3a. Based on hardware components, hTetro can transform to seven different morphologies described in Figure 3b and named as O, Z, L, T, J, S, I shapes.

## 3. On-Board-LiDAR-Sensor hTetro ROS Based System

**Figure 4:**
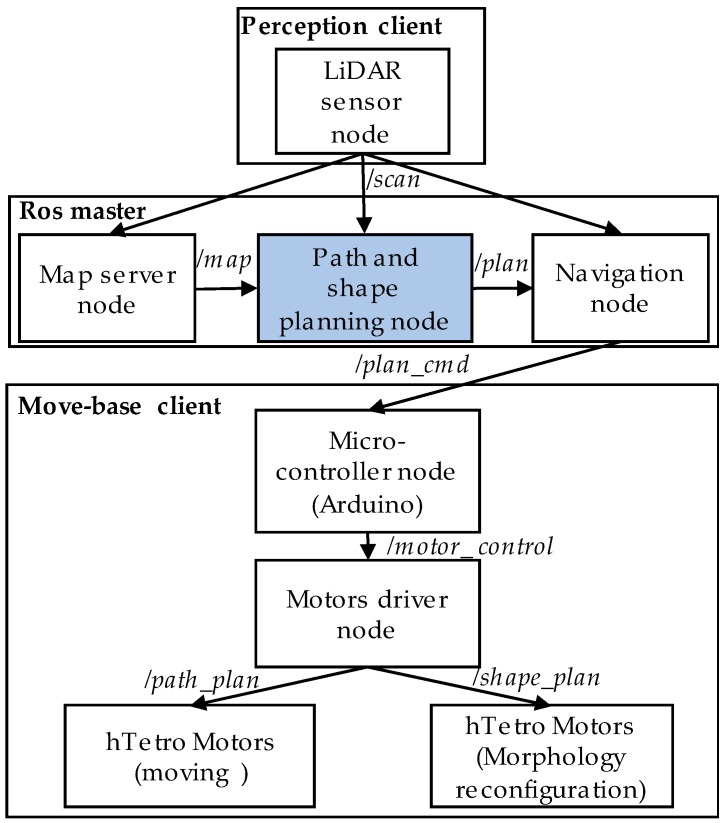
ROS based system of hTetro.

The proposed system is built on the ROS platform [23]. ROS provides the infrastructure and mechanism for ROS modules playing the roles as ROS nodes to communicate and control the hTetro hardware modules by ROS topics, ROS messages and ROS services. The ROS-based block diagram of the system is shown in Figure 4. The ROS master installed on Intel computer stick monitors the entire ROS system. Based on /*scan* topics of LiDAR sensor node and prebuilt/map topics of map server node, path and shape planning node generates the /*plan* topics. Using this */plan* topics, a navigation node was created to achieve the smooth locomotion inside the prebuilt map. This ROS node will create plan commands denoted as /*plan_cmd* and sends to Arduino controller. After receiving */plan_cmd*, Arduino controls motor driver node by */motor_control* topics. The hTetro moving and morphology reconfiguration is based on */path_plan* and */shape_plan* topics, respectively.

**Figure 5:**
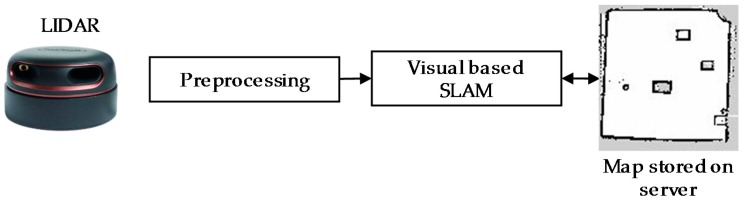
Mapping processes.

**Figure 6:**
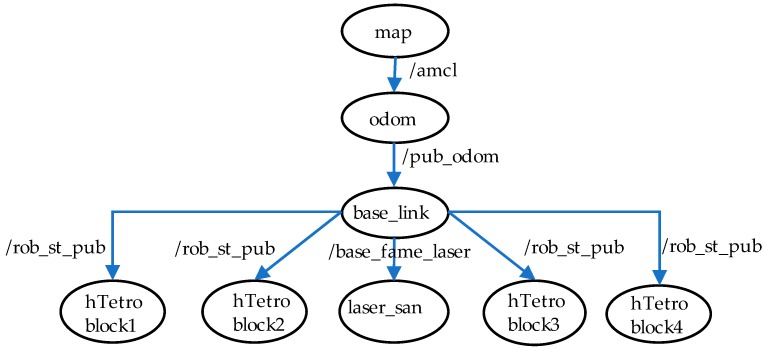
Transformation frame (TF) transformation tree.

To navigate autonomously in ROS system, a map of the robot environment should be built. SLAM algorithm can do the mapping processes as Figure 5. Many SLAM methods have been proposed to achieve the purpose of building the map of robot environment. The first approach is based on monitoring the real-time position of the robot which is placed at any location of the environment during the map building process. The most commonly used method for this approach is the use of Gmapping [24,25]. Odometry values that estimate the position of the robot can be obtained either by using data provided by the combination of a good sensor and a good computational algorithm or by the fusion of multiple sensors such as IMU, GPS or Encoders used in the motors. In most of the cases the robot pose in the Odometry frame gets drifted over the long run this is because of hardware defects and sensor noise, so it is not always advised to rely on the single sensor to estimate the robot pose. A filter algorithm such as adaptive Monte Carlo localization (AMCL) [26] can refine the Odometry information and maintain the relationship between the coordinates of global map and local map, Odometry, base link and robot block module frames in ROS systems. AMCL is the technique that uses particle filter in real-time filter out the noise in Odometry to estimate a more accurate position of the robot in the environment. In many robot platforms, the Odometry information is often difficult to compute accurately if it relies on the data provided by the wheel encoder because of the wheel slippage issues. To overcome this challenge, another approach that uses the high-speed and large range of view sensors to estimate and maintain the robot pose by matching the features of the positions derived from sensor data when the robot moves around the unknown area. The information about the translation, rotation and velocity of Odometry can be derived by using the feature detection and matching techniques. This approach is very similar to the construction of a panoramic view where multiple partially overlapping view images are assembled to produce a large field of view image. One advantage of this method is that the real-time position information of the robot can be estimated from the matching features of visual sensor data without even depending on wheel encoders or imu sensors that often shows errors due to wheel slippage or interference in the external magnetic field. The disadvantage of this approach is that it requires a good quality laser sensor and the sophisticated real-time processes to detect the similarities between frames. Recently, laser sensors with the high scanning rate wide field of view LIDAR and robust feature matching techniques make this approach more simple and effective in robot pose estimation. It is worth to note that the hTetro has the ability of self-configuring to other morphologies and changing the moving direction to opposite direction without the need for pivot turn as other robots. Determining the odometry data of hTetro by computing the values from the wheel encoder is more complicated because of the fusion of data from all the 16 wheels and thus the probability of getting an error value is almost 16 times to the normal two-wheeled robots. The complication level increases more when the robot does the reconfiguration. In this paper, we use the rpLidar laser sensor to scan and built the map of the robot environment as in Figure 5. Hector mapping [27] and laser scan matcher [28,29] techniques are used to compute the odometry of the robot. These methods use the concept of finding the similarities between the new frame and the previous frames that the robot has passed to estimate robot Odometry information. After applying an AMCL to refine the location, the ROS transformation package will maintain the relationship between positions of the four hTetro blocks frames, laser_scan frame, local base_link frame, local Odometry frame and global map frame. The transformation frames (TF) tree of the system is shown in Figure 6.

After building the map of the robot environment by using the hector mapping, the maps are saved by using the map server service of ROS to leverage the ability of the autonomous navigation. The saved map includes two files, an image file with Pym format and a configuration file with XML format. The image file defines the pixel values corresponding to the specific elements in the real environment. The space pixel is defined based on the map resolution in ROS system. ROS considers the prebuild map as an image. Pixels resolution equals to grid resolution in this paper. In particular, for each pixel location (x,y) in the prebuild map, the pixel value M(x,y)=0 corresponds to the free space pixel, M(x,y)=100 represents for obstacle pixel or the boundary of the room pixel and M(x,y)=−1 corresponds to the undefined pixel where the laser cannot be scanned (Figure 4). The information in the configuration file describes the origin $\left(x_{o},y_{o}\right)$ position, map scale $M_{s}$, map width $M_{w}$ and map height $M_{h}$. The x-axis and y-axis go along the width and height of the map, respectively. From the information in the configuration file, Equations (1) and (2) are used to switch between the pixel location (x,y) on the image file and its coordinate (ℂx,ℂy) in the real environment.

(1) $$\mathbb{C}x=x_{0}+(x-M_{w}/2)M_{s},$$

(2) $$\mathbb{C}y=y_{0}+(y-M_{h}/2)M_{s},$$

The pre-built map is subdivided into a grid of predefined size of 0.1 m squares. One square unit consists of its location at (x,y) and its length. The conversion of pixel coordinates to the actual position coordinates in m will be done using Equations (1) and (2). The distance between two adjacent cells is the minimum distance of the robot moving step in this paper.

## 4. Proposed Path and Shape Planning Method

### 4.1. A-Star Based-Zigzag Planning

**Figure 7:**
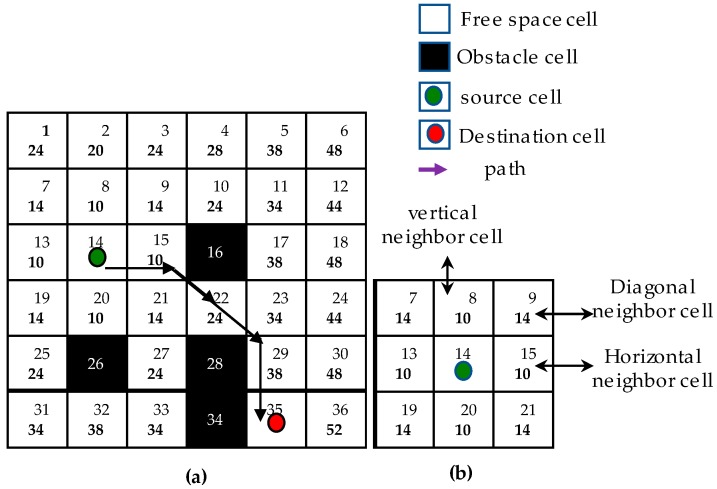
A-star shortest path searching. (**a**) Estimating A-star path, (**b**) neighboring cells.

**Figure 8:**
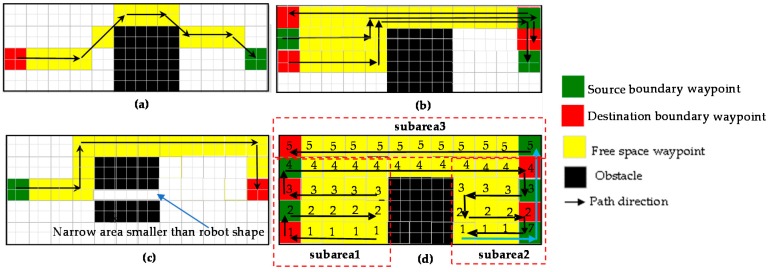
Drawbacks of conventional A-star based methods for area coverage. (**a**) A-star with the diagonal movement, (**b**) A-star for area coverage, (**c**) uncovered narrow space by fix morphology, (**d**) subareas A-star based area coverage [8].

The final goal of solving area coverage problems is to cover the free space areas defined in the prebuilt map by navigating automatically and following the predefined path created by the global planner. To this end, numerous methods have been proposed. The conventional offline methods select the waypoints manually with the knowledge of maps shapes and sizes as well as obstacles shapes and positions to create the area coverage path. Besides, several cleaning robots use bump sensors to detect and follow the obstacle boundaries to form area coverage path. A-star algorithm [22] is the most popular and widely used method to compute and plan the path for the robot to navigate autonomously by avoiding the obstacles. The main idea of this algorithm is to find the shortest path between the starting point and the destination point based on the cost function. A-star algorithm is based on a grid map. The grid cells are categorized into source cell, destination cell, free space cells and obstacle cells. Figure 7a describes one such example of the algorithm to find the shortest path from cell 14th to cell 35th. There are eight neighboring cells around the cell in grid map as Figure 7b. In the first step of A-star, each neighboring free space cell around the source cells is assigned a corresponding cost such as horizontal and vertical neighboring cells have the cost of 10 while the diagonal neighboring cells have the cost of 14. The neighboring obstacle cells do not have any cost. Then the cost of these free space neighbors will be accumulated until the destination cell is reached. The shortest path is selected by tracing back to find the cells with the smallest value from destination cell to source cell. In the case of maximizing area coverage with the ability to avoid obstacles, zigzag scanning-based A-star approach can be used, by defining a set of waypoints locations at the boundary as in the zigzag pattern, then letting the A-star to compute the shortest path to clear these waypoints. To cover the entire area defined by the boundary of the room on the map, the trajectory is a zigzag line which is made up of evenly spaced segments. The end point of each segment is connected to the starting point of the next segment. The conventional algorithm A-star has the limitations of not covering the free space cells in the defined range and revisiting free space cells that have been already covered. Assuming that the robot width equals the length of 2 cells, the drawbacks of the A-star algorithms are depicted in Figure 8a–c. It can be observed in Figure 8a that the diagonal moving feature of A-star making the shorter path from source to destination is triggered with which some cells around the diagonal trajectory will not be covered. In Figure 8b, if an obstacle appears on the path connecting two waypoints, there are two options to choose the path in order to avoid obstacles by going up or going down. If A-star algorithm opts to move upon reaching the obstacle cells as in Figure 8b, some cells below the obstacle remain uncovered and the covered cells above the obstacles will be revisited to find the way connecting from right to left boundary waypoint. Similarly, the upper cells will not be covered and the under cells are revisited if A-star decides to go down on reaching obstacle cells. Moreover, the A-star algorithm selects a path that goes around free space defined as narrow space pixels as depicted in Figure 8c.

The modified A-star based-zigzag scanning method for the area coverage problem has been proposed in the research of [30]. In this method, the map is divided into the sub-maps bordered by obstacle boundary. Then these subareas are covered by zigzag planning. After finishing each sub-area, the algorithm chooses the next uncovered area yielding the shortest distance with the current covered one. A-Star algorithm will be used to find the path to this uncovered area; this path is called way-out path as in Figure 8d. However, there are some limitations in this algorithm. Firstly, the algorithm is considered for the obstacles with relatively simple shapes. Secondly, when moving to the next uncovered sub-map, if the last covered cell of a sub-map is located at a place where there are no neighboring cells from the next uncovered sub-map some cells of current subarea needs to be revisited to reach the uncovered subarea. The paths are represented by blue arrows in Figure 8d. Finally, the problem of covering the narrow space constrains is not solved in this method. This problem is inevitable for the fixed morphology robots which cannot change its morphology.

**Figure 9:**
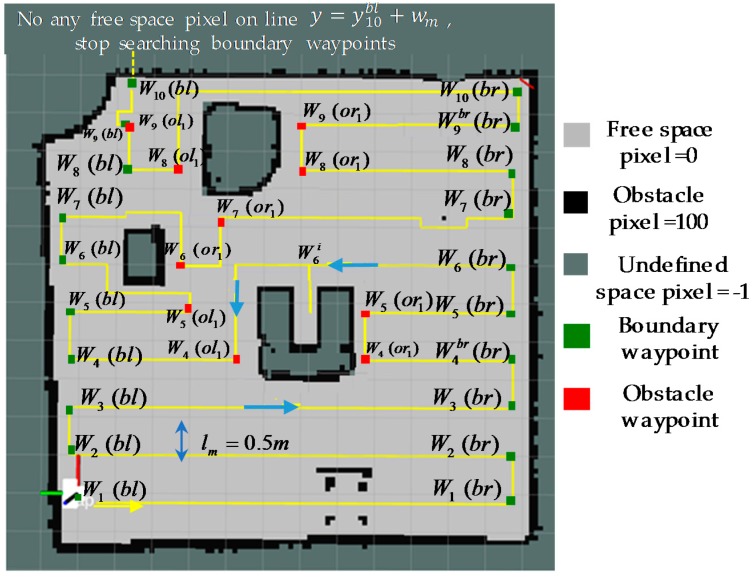
Boundary waypoints and obstacle waypoints.

In this paper, a modified A-star based zigzag scanning is proposed to plan trajectory including a set of intermediate waypoints for a Tetris inspired self-reconfigurable robot hTetro. Specifically, the shapes and dimensions of the maps generated by SLAM algorithm in ROS environment can be set randomly or acquired dynamically in real time. Consequently, the areas needed to be covered and obstacle characteristics such as shapes and locations as well as map border are not known before creating the coverage paths. The proposed method uses features of the maps to determine the different types of waypoints including boundary waypoints, obstacle waypoints autonomously, Furthermore, unlike the traditional path planning defies only waypoints locations, the waypoints in the proposed method include both accurate locations and appropriate morphology in hTetro robot. As per the results the final objective of covering maximum areas, minimizing the issue of revisiting the areas that have been covered and the ability to navigate through narrow space constraints of the maps built on ROS system can be accomplished efficiently. The process of creating a zigzag path planning for a map is depicted in Figure 9. The trajectory represented in Figure 9 is set to start at the first waypoint and then follows the yellow path towards the last waypoint. The first waypoint is defined as the origin point located at the bottom left corner. Note that the x-axis is denoted green and the y-axis is denoted red. Since the obstacles locating randomly in the map have the arbitrary shapes and the map are segmented into grids, the grid-based A-star algorithm is used to find the shortest path to avoid the obstacles and connect each pair of auto-generated waypoints. Movement in diagonal direction in the A-star algorithm is disabled to ensure the maximum area coverage.

**Figure 10:**
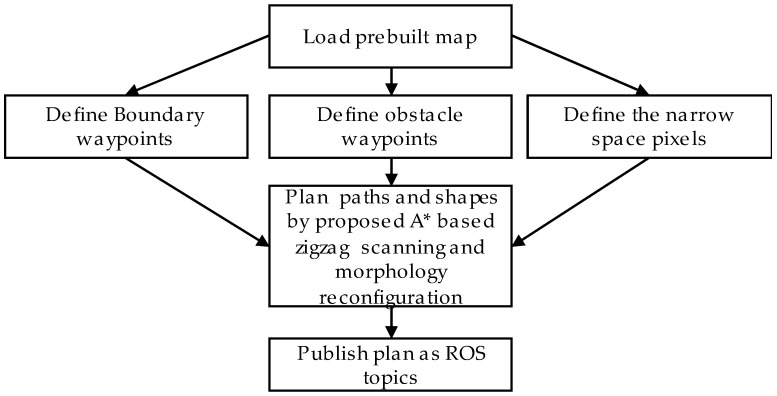
Global path and shape planner flowchart.

The boundary waypoints marked as the green and obstacle waypoints marked as and red cells in Figure 9. Boundary waypoints and obstacles waypoints staying near the border of the map and obstacles respectively are the locations where zigzag scanning segments are terminated. The procedures of determining in a random map these waypoints are detailed in next sections. After the global shape and path planner has autonomously defined the waypoints, the hTetro will follow the path and perform shapeshifting that are necessary to avoid obstacles, pass through the narrow spaces and clear the waypoint. The flowchart depicted in Figure 10 describes the algorithm to identify the locations and the morphologies at intermediate waypoints to adapt to particular situations.

New zigzag scanning pattern is proposed to enable the global planner in finding the paths that cover maximum free space area of the prebuild maps and reduce the issue of revisiting the already covered cells. Specifically, the global planning algorithm defines a range of {$\left\{W_{n}^{}(k)\{(x_{n}^{k},y_{n}^{k}),mo_{n}^{k}\}\right\}$ and $\left\{W_{n}^{i}\{(x_{n}^{i},y_{n}^{i}),mo_{n}^{i}\}\right\}$, where a set of $\left\{W_{n}^{}(k)\right\}$ is the boundary or obstacle waypoints and a set of free space waypoints $\left\{W_{n}^{i}\right\}$ with value *i* starting from number 1 is used to connect a set of $\left\{W_{n}^{}(k)\right\}$. The *n* represents the horizontal line $y=y_{n}$ on map frame coordinate where the waypoints stay on. The successive waypoints $W_{n}^{i}$ that the distance between two adjacent waypoints on the horizontal or vertical direction has the distance of one grid square will route the waypoints $W_{n}^{}(k)$ to create the complete path trajectory of unbroken zigzag line. The components of one $W_{n}^{}(k)$ include waypoint location $\left(x_{n}^{k},y_{n}^{k}\right)$ and waypoint morphology plan $mo_{n}^{k}$. Note that $k=\{bl,br,ol_{i},or_{i}\}$ where $b_{l},b_{r},o_{l}^{i},o_{r}^{i}$ are boundary waypoint on the left side of map border, boundary waypoint on the right side of map border, waypoint on the left side of obstacle number *i*, waypoint on the right side of obstacle number *i* respectively on the horizontal line $y=y_{n},$. For each morphology $m_{n}^{k}$ with the robot size in pixel unit defined as $w_{m}\times h_{m}$ (or $l_{m}\times d_{m}$ in meter unit after being converted by Equations (1) and (2)), the dimension which is perpendicular to the heading direction of the robot is defined as $w_{m}$ and the $h_{m}$ is the remaining dimension. As shown in Figure 3c,d for the same I configuration but $w_{m}$ is defined differently for horizontal and vertical moving directions. Unlike fix morphology robot, the width size of hTetro can be reduced by haft by changing the morphology such as from O to I during vertical moving or from I to O during horizontal moving. The length of cells which are occupied by a specific $m_{n}^{k}$ of robot, width is defined as same as $w_{m}$. In this paper, the default morphology is O shape with *l_m_* = 0.5 m and *d_m_* = 0.5 m The I shape has the *l_m_* = 0.25 m and *d_m_* = 1 m The first waypoint $W_{0}^{}(bl)$ is the origin of the map. The process of finding intermediate waypoints will start from the first waypoint $W_{0}^{}(bl)$. Note that, $Μ(x_{n}^{k},y_{n}^{k})$ is the pixel value of the location $\left(x_{n}^{k},y_{n}^{k}\right)$.

### 4.2. Boundary Waypoints Detection

**Figure 11:**
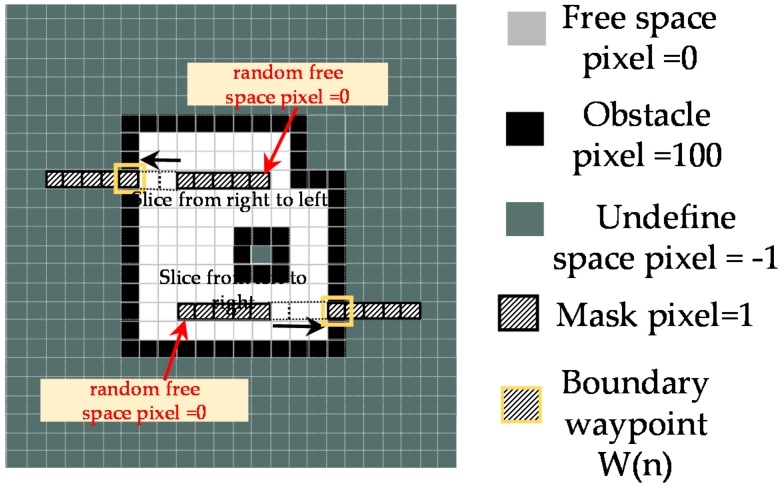
Left and right boundary waypoints estimating.

The following Algorithm 1 will be used to find waypoints autonomously at the boundary of a pre-built map with random sizes and dimensions. Assuming the next step of the algorithm is to determine the waypoint $W_{n}(br)$ staying on the right side of the known waypoint $W_{n}(bl)$. Since they are on the same horizontal line, these two waypoints locate on $y=y_{n}^{}$ and $y_{n}=y_{n}^{bl}=y_{n}^{br}$. A filter mask Φ of 1×m with a width of 1 pixel, the length of *m* pixels and the value of pixels equal to 1 is created as Figure 11. The free space pixel at $\left(x_{n}^{bl}+r,y_{n}^{bl}\right)$ on the map, where $M(x_{n}^{bl}+r,y_{n}^{bl})=0$ and *r* is random integer value such as $0\leq x_{n}^{bl}+r\leq M_{w}$ is selected. The first pixel Φ(1,1) of the mask is set at this pixel location. The mask slides with the step of one pixel from this location to the right and filters each visited pixel $\left(x_{n}^{bl}+r+i,y_{n}^{bl}\right)$ on the line $y=y_{n}^{bl}$ of the map as Figure 11. At each location, the Equation (3) is used to calculate the filtered value $f_{w}(x_{n}^{bl}+r+i,y_{n}^{bl})$ of the mask with a same mask size partition on the map. It is worth to note that in a map created by ROS, the pixels on the right side of the map right boundary pixel have the same value of −1. Similarly, all the pixels on the left side of the map left boundary pixel have the same value of −1. As the results, the $f_{w}(x_{n}^{bl}+r+i,y_{n}^{bl})$ will equal -*m* if the partition at to-be-filtered pixel stays entirely on the undefined areas of the map. If the value $f_{w}(x_{n}^{bl}+r+\hat{i},y_{n}^{bl})=100th-m-1$, where value $\hat{i}$ can be found by Equation (4) and *th* is the parameter representing the pixel thickness of the border of the map (*th* equals 3 pixels in this paper), then the location $\left(x_{n}^{bl}+r+\hat{i},y_{n}^{bl}\right)$ in the range $\left[(x_{n}^{bl}+r),M_{w}\right]$ will correspond to the right boundary pixel on the line $y=y_{n}^{bl}$ of the map. The value of m is chosen so that the length of the mask is sufficiently larger than the biggest value of the lengths of the obstacles because the empty spaces inside the objects boundaries where the Lidar scan is blocked are also undefined spaces and have a value of −1. In this paper, m is set to 100 pixels. The right boundary waypoint on $W_{n}^{}(br)$ will have the coordinate $\left(x_{n}^{br},y_{n}^{br}\right)$, where $x_{n}^{br}=x_{n}^{bl}+r+\hat{i}-w_{m}/2$, $y_{n}^{bl}=y_{n}^{br}$. Since the rpLidar is fixed at the block 2 of hTetro, the value $w_{m}/2$ where $w_{m}$ is the robot width in pixel is added to make the safety distance with border. The processes are the same for the case where the mask slides from right to left for determining the pixel boundary on the right side.

(3) $$f_{w}(x_{n}^{bl}+r+i,y_{n}^{bl})=\sum_{1\leq q\leq m}M(x_{n}^{bl}+r+i(sig)q,y_{n}^{bl})\Phi (1,q),$$

where sig=1 to find the boundary pixel on left sig=−1 to find the boundary pixel on the right

(4) $$\hat{i}=i|0\leq i\leq M_{w}-x_{n}^{bl}-r\;and\;f_{w}(x_{n}^{bl}+r+i,y_{n}^{bl})=100th-1-m,$$

**Algorithm 1** Determination of boundary waypoints1: **function** GetLeftBoundayWaypoints(map *M* the origin $\left(x_{o},y_{o}\right)$, threshold th, *m*, hTetro width $w_{m}$)2: **initial**
$x_{n}^{bl}=x_{o}$, $y_{n}^{bl}=y_{o}$, select random pixel $\left(x_{n}^{bl}+r,y_{n}^{bl}\right)$, *r:* random value3: **while** pixel $\left(x_{n}^{bl}+r,y_{n}^{bl}\right)$ is the free space pixels $M(x_{n}^{bl}+r,y_{n}^{bl})=0$
**do**4:   i←05:   filter the pixel $\left(x_{n}^{bl}+r+i,y_{n}^{bl}\right)$ with the filter mask Φ length *m*, value = 16:   **while** number of undefined pixels in range $\left[(x_{n}^{bl}+r+i,y_{n}^{bl}),(x_{n}^{bl}+r+m+i,y_{n}^{bl})\right]$
≤m−1−th
**do**7:     i←i−18:   the right boundary waypoint $W_{n}^{}(bl)\leftarrow (x_{n}^{bl}+r+i+w_{r}/2,y_{n}^{bl})$9: **end while**10: **function** GetRightBoundayWaypoints(map *M*, left boundary waypoint $\left(x_{n}^{bl},y_{n}^{bl}\right)$, threshold th, *m*, hTetro width $w_{m}$)11:   **initial**
$x_{n}^{br}\leftarrow x_{n}^{bl}$, $y_{n}^{br}\leftarrow y_{n}^{bl}$, select random pixel $\left(x_{n}^{br}+r,y_{n}^{br}\right)$, *r:* random value12:   **while** pixel $\left(x_{n}^{br}+r,y_{n}^{br}\right)$ is the free space pixels $M(x_{n}^{br}+r,y_{n}^{br})=0$
**do**13:    *i*←*0*14:    filter the pixel $\left(x_{n}^{br}+r+i,y_{n}^{br}\right)$ with the filter mask Φ length *m*, value = 115:    **while** number of undefined pixels in range $\left[(x_{n}^{br}+r+i,y_{n}^{br}),(x_{n}^{br}+r+m+i,y_{n}^{br})\right]$
≤m−1−th
**do**16:      i←i+117:    the left boundary waypoint $W_{n}^{}(br)\leftarrow (x_{n}^{br}+r+i-w_{r}/2,y_{n}^{br})$18:   **end while**19:   **end while**20: **end function**GetRightBoundayWaypoints21:   increase the y coordinate $y_{n}^{bl}\leftarrow y_{n}^{bl}+w_{m}$22: **end while**23: **return** left boundary waypoints, right boundary waypoint24: **end function**GetLeftBoundayWaypoints

Once the right and left boundary waypoints on the $y=y_{n}^{l}$ line have been determined, the Algorithm 1 will continue to identify the two similar boundary waypoints on the line $y=y_{n}^{bl}+w_{m}$. The same mechanism described in the previous section is applied to find the left and right boundary waypoints on this line. After increasing the value y of the current boundary waypoint, if it is impossible to select any random point having the pixel value $M(x_{n}^{bl}+r,y_{n}^{bl})=0$ on the line $y=y_{n}$ of the map, the process of searching boundary waypoints will stop. If this condition happens, the previous boundary waypoint stays at the uppermost boundary of the map and it is identified as the last boundary waypoint to complete the zigzag trajectory as Figure 9.

### 4.3. Obstacle Waypoints Detection

After defining boundary waypoints, the global planner will auto-define the waypoints near the obstacles in map according to Algorithm 2. Note that the obstacles locating randomly in the map have the arbitrary shapes. Specifically, on the horizontal line $y=y_{n}^{bl}$ which connects $W_{n}^{bl}$ and $W_{n}^{br}$ the location from the first free space pixel on the left side of obstacle pixel with a distance of $w_{m}/2$ will be selected as the location of left obstacle waypoint $W_{n}^{}(ol_{i})$ and the location from the first free space pixel on the right side of obstacles with a distance of $w_{m}/2$ will be defined as the location of right obstacle waypoint $W_{n}^{}(ol_{i})$.

**Algorithm 2** Determination of obstacle waypoints1: **function** GetLeftObstacleWaypoints(map *M*, boundary waypoints)2: **if** there is no right obstacle waypoint on line $y=y_{n}^{bl}$
**then**3:   **initial**
$W_{n}^{}(ol_{i})\leftarrow (x_{ni}^{ol},y_{ni}^{ol})$, $x_{ni}^{ol}\leftarrow x_{n}^{bl}$, $y_{ni}^{ol}\leftarrow y_{n}^{bl}$, i←04: **else**5:   **initial**
$W_{n}^{}(ol_{i})\leftarrow (x_{ni}^{ol},y_{ni}^{ol})$, $x_{ni}^{ol}\leftarrow x_{n(i-1)}^{or}$, $y_{ni}^{ol}\leftarrow y_{n(i-1)}^{or}$, i←06: **end if**7: **while** pixel $\left(x_{ni}^{ol}+i,y_{ni}^{ol}\right)$ is the free space pixel $M(x_{ni}^{ol},y_{ni}^{ol})=0$
**do**8:   i←i+19: **end while**10: **if**
$x_{ni}^{ol}+i$ < $x_{n}^{br}$
**then**11:   the left obstacle waypoint $W_{n}^{}(ol_{i})\leftarrow (x_{ni}^{ol}+i-w_{r}/2,y_{ni}^{ol})$12: **elseif** there is no right obstacle waypoint on line $y=y_{n}^{bl}$
**then**13:   no left obstacle waypoint on line $y=y_{n}^{bl}$14: **end elseif**15: **end if**16: **function** GetRightObstacleWaypoints(map *M*, boundary waypoints, left obstacle waypoint $W_{n}^{}(ol_{i})$)17: **initial**
$W_{n}^{}(or_{i})\leftarrow (x_{ni}^{or},y_{ni}^{or})$, $x_{ni}^{or}\leftarrow x_{ni}^{ol}+w_{r}/2$, $y_{ni}^{or}\leftarrow y_{n}^{bl}$, i←018:   **while** pixel $\left(x_{ni}^{or}+i,y_{ni}^{or}\right)$ is the obstacle pixel $M(x_{ni}^{ol},x_{ni}^{or})=100$ or obstacle pixel $M(x_{ni}^{ol},x_{ni}^{or})=-1$
**do**19:     i←i+120:   **end while**21:   **if**
$x_{ni}^{or}+i$ < $x_{n}^{br}$
**then**22:     the right obstacle waypoint $W_{n}^{}(or_{i})\leftarrow (x_{ni}^{or}+i+w_{r}/2,y_{ni}^{or})$23:   **elseif** there is no left obstacle waypoint on line $y=y_{n}^{bl}$
**then**24:     no right obstacle waypoint on line $y=y_{n}^{bl}$25:   **end elseif**26: **end if**27: **end function** GetRightObstacleWaypoints28: **return** left obstacle waypoints, right obstacle waypoints29: **end function** GetLeftObstacleWaypoints

### 4.4. Proposed A-Starbased Path Planning Strategy

**Figure 12:**
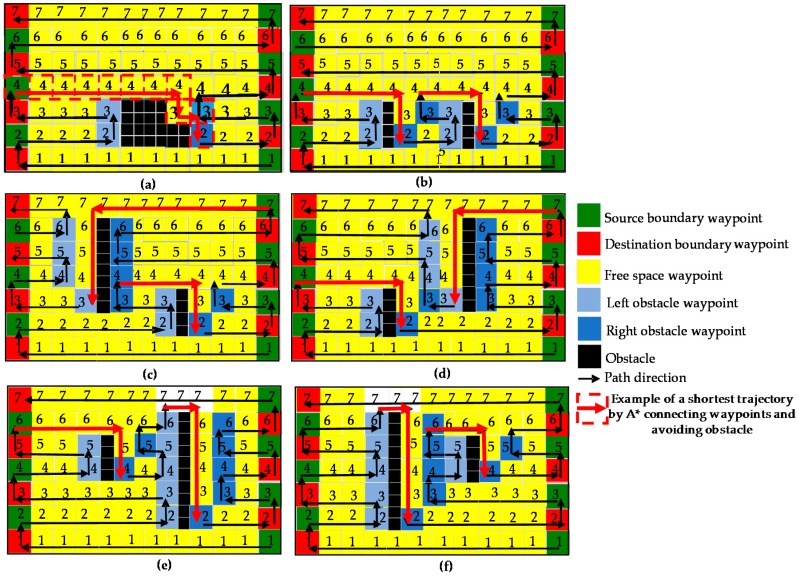
Proposed zigzag scanning method. (**a**) the single obstacle, (**b**) obstacles with the same size and locate on same lines, (**c**–**f**) obstacles with different sizes and locate on different lines between left and right boundary waypoints.

Each segment of the zigzag pattern of the path will be created by adding a set of $W_{n}^{i}$ to connect any two, boundary and obstacle waypoints with these two waypoints are the start and end points of this segment. In particular, if there is no any obstacle waypoint on the line $y=y_{n}^{bl}$ connecting two boundary waypoints $W_{n}(bl)$ and $W_{n}(br)$ as cells marked 1, 5, 6, 7 in Figure 12a the global planner adds set of cells $W_{i}^{}$ along the line $y=y_{n}^{bl}$ and generating by A-star path searching to connect these two waypoints. The path generated by A-star is same as zigzag scanning. In the cases of existing obstacle between waypoints at cells 2, 3, 4 in Figure 12a, firstly the path planning creates zigzag lines to cover the left boundary waypoints and the left obstacle waypoints. Specially, assuming that at present, zigzag trajectory moves from left to right and meet the first obstacle, the height of the obstacle is estimated by the following condition. From the obstacle pixel, the set of obstacle pixels of this obstacle object in a known map can be found by finding the linking neighboring pixels, which have the pixel value of 100. The value corresponding to the highest y value denoted as $y_{o}^{h}$ is set to the height of this obstacle object. The range of left and right boundary waypoints having y value in a range $\left[0:M_{w},y_{n}^{bl}:y_{o}^{h}+w_{m}\right]$ denoted at [$W_{n}(bl):$$W_{n+o}(bl)$] and [$W_{n}(br)$:$W_{n+o}(br)$]. The range of left and the right obstacle waypoints having the *y* coordinate value in the range of $\left[0:M_{w},y_{n}^{bl}:y_{o}^{h}+w_{m}\right]$ are also identified and denoted as [$W_{n}(ol_{i}):$ $W_{n+o}(ol_{i})$] and [$W_{n}(or_{i})$:$W_{n+o}(or_{i})$] with *s* is the obstacle number *i.* The $w_{m}$ is added to make the safety distance which ensures the planned path can avoid the obstacle. After the range of obstacle waypoints have been indented, instead of going to boundary waypoints $W_{n}(br)$ from the boundary waypoint $W_{n}(bl)$ by the conventional A-star algorithm, the zigzag scanning technique will be applied to clear the area defined by waypoints [$W_{n}^{}(bl)$:$W_{n+o}^{}(bl)$] and $\left[W_{n}(ol_{1}):W_{n+o-1}(ol_{1})\right]$. The scanning order by A-star path searching will execute from left waypoint to right waypoint then move to upper right waypoint to scan left waypoint on the same line until the last waypoint has been cleared as in Figure 12a. After creating zigzag lines to clear left boundary waypoints and left obstacle waypoints, then the shortest trajectory including grid cells with red arrows as in Figure 12a generated by A-star connects the leftmost boundary waypoint $W_{n+o}^{}(bl)$ marked as a green cell with number 4 to the first right obstacle waypoint $W_{n}(or_{1})$ marked a blue cell with number 2. As one can observe, the shortest path by A-star will follow the obstacle boundary cells. If there is another $W_{n}ol_{i+1}$ of obstacle number *i* + 1 located on the right side of $W_{n}or_{i}$, the same zigzag scanning strategy will clear the area defined by waypoints $\left[W_{n}(or_{i}):W_{n+o-1}(or_{i})\right]$ and $\left[W_{n}(ol_{i+1}):W_{n+o-1}(ol_{i+1})\right]$. In case if there is no $W_{n}ol_{i+1}$, the final segment defined by waypoints $\left[W_{n}(or_{i}):W_{n+o}(or_{i})\right]$ and $\left[W_{n}(br_{}):W_{n+o}(br_{})\right]$ will be covered by the same zigzag scanning strategy as Figure 12a. Note that, to reduce the problem of revisiting the cells at the right side of obstacle boundary when zigzag scanning moves from left to right, the right obstacle waypoint from $W_{n+1}(or_{1})$ to $W_{n+o}(or_{1})$ is shifted to the right by the number of cells equal to robot width $w_{m}$ by assuming the width between obstacle *i* and obstacle *i* + 1 is larger than $2\times w_{m}$. The right waypoint is shifted and then marked as blue cells with number 3 in Figure 12a. A similar mechanism is applied in the case of zigzag scanning that moves from the right to left, the left obstacle waypoint from $W_{n+1}(ol_{1})$ to $W_{n+o}(ol_{1})$ is shifted to the left by the number of cells equal to robot width $w_{m}$.

The Figure 12b presents zigzag scanning for a case where the obstacles have the same size and stay on the same horizontal lines. The Figure 12c–f describes the cases where obstacles have different size and stay on different horizontal lines. As one can see the path generated by global planer cover all cells and does not have any revisited cells in cases of Figure 12a–d and revisit several cells marked as white color and number 7 in cases of Figure 12e,f.

### 4.5. Narrow Spaces Detection and Covering

Covering the narrow spaces involves significant challenges for the robots with fixed morphology. When this scenario come to hTetro, it can move through narrow spaces effectively by changing its morphology. The I and O shapes of hTetro are used to demonstrate the transformation ability of hTetro to cover the narrow space and build the path planning. The narrow space is defined so that its width is smaller than current hTetro morphology width $w_{m}$. Note that the fix morphology robots consider the narrow spaces are the parts the solid obstacles that make it impossible to cover areas over narrow spaces. By identifying narrow spaces in random map, if the current location of the zigzag path offsets the distance of the current robot width $w_{m}$ with the nearest narrow space, the hTetro morphology with the width size smaller than narrow space width is assigned to the shape plan. After changing to morphology with the smaller width, hTetro will consider the narrow space is the typical free space. Then the path created by A-star is added to path planner will guide the robot to cover over the narrow space.

**Figure 13:**
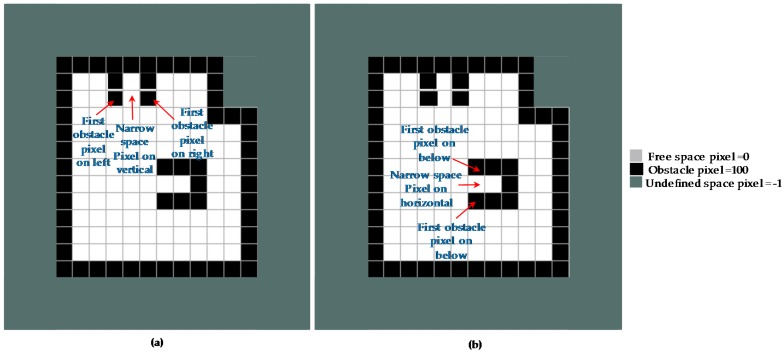
Narrow space pixels estimation. (**a**) Defining horizontal vertical narrow pixel, (**b**) defining horizontal narrow pixel.

To navigate through narrow space constraints, the global shape planner must possess a capability of identifying narrow areas in the map for each specific robot configuration as Figure 13. An Algorithm 3 for determining the narrow space areas in the entire prebuilt map is presented as follows. Specifically, to find the narrow space pixels on the vertical direction, considering one free space pixel (x,y) with value M(x,y)=0 on the map, the locations of the first obstacle pixel with value = 100 on the left and the first obstacle pixel with value = 100 on the right this free space pixel is defined and denoted as $\left(x+v_{l},y\right)$ and $\left(x-v_{l},y\right)$ respectively. If the value of $v_{l}+v_{r}$ is lower than a narrow space threshold $nth_{m}^{}$ and larger than $0.5\times nth_{m}^{}$ with *m* representing a specific hTetro morphology as in Equation (5), this free space pixel is set to belong in a narrow space area. The value $nth_{m}^{}$ is chosen so that it is smaller than the width $w_{m}$ of the robot. In this paper, we set $nth_{m}=0.8w_{m}$. The similar algorithm as in Equation (6) can be applied to find the narrow space pixel on the horizontal direction where, $h_{a}$ is the offset value on the above side and $h_{b}$ is the offset value on the below side. The narrow pixel is denoted as $\left(x_{ns},y_{ns}\right)$ and the narrow space pixel values $M(x_{ns},y_{ns})$ will be assigned a value of 200 to distinguish them from other pixels of the map.

(5) $$\begin{array}{l} 0.5\times nth_{m}^{}<v_{l}+v_{r}<nth_{m}^{},\\ where\;M(x,y)=0,\;M(x+v_{l},y)=100,\;M(x-v_{l},y)=100 \end{array}$$

(6) $$\begin{array}{l} 0.5\times nth_{m}^{}<h_{a}+h_{b}<nth_{m}^{},\\ where\;M(x,y)=0,\;M(x,y+h_{a})=100,\;M(x,y-h_{b})=100 \end{array}$$

**Algorithm 3** Determination of narrow space areas1: **function** GetVerticalNarrowSpace(map *M*, threshold $nth_{m}$)2: **For** all pixel (x,y) in map M
**do**3: **if** the pixel (x,y) is the free space pixel M(x,y)=0
**then**4:   $v_{l}\leftarrow 0$, $v_{r}\leftarrow 0$5:   **while** pixels $\left(x-v_{l},y\right)$, $\left(x+v_{r},y\right)$ are the free space pixel **do**6:     $v_{l}\leftarrow v_{l}+1,\;v_{r}\leftarrow v_{r}+1$7:   **end while**8:   **if**
$0.5\times nth_{m}^{}<v_{l}+v_{r}<nth_{m}^{}$
**then**9:     narrow pixel $\left(x_{ns},y_{ns}\right)$ with $x_{ns}\leftarrow x,y_{ns}\leftarrow y$10:      $M(x_{ns},y_{ns})\leftarrow 200$11:   **end if**12: **end if**13: **function** GetHorizontalNarrowSpace(map *M*, threshold $nth_{m}^{}$)14:   **for** all pixel (x,y) in map M
**do**15:   **if** the pixel (x,y) is the free space pixel M(x,y)=0
**then**16:     $h_{a}\leftarrow 0$, $h_{b}\leftarrow 0$17:     **while** pixels $\left(x,y-h_{b}\right)$, $\left(x,y+h_{a}\right)$ are the free space pixel **do**18:       $h_{a}\leftarrow h_{a}+1,\;h_{b}\leftarrow h_{b}+1$19:     **end while**20:     **if**
$0.5\times nth_{m}^{}<h_{a}+h_{b}<nth_{m}^{}$
**then**21:       narrow pixel $\left(x_{ns},y_{ns}\right)$ with $x_{ns}\leftarrow x,y_{ns}\leftarrow y$22:       $M(x_{ns},y_{ns})\leftarrow 200$23:     **end if**24:   **end if**25: **end function** GetHorizontalNarrowSpace26: **return** narrow pixels27: **end function** GetVerticalNarrowSpace

Once the narrow space pixels have been determined, the global planner will start its process of finding the intermediate $W_{n}^{i}$ waypoints passing through the narrow spaces connecting between boundary waypoint $W_{n}(bl)$ and boundary waypoint $W_{n}(br)$. The path planning algorithm will decide the path by finding the nearest narrow pixel with the current waypoint $W_{n}^{i}$. In order to archive it, the narrow space pixel in a window Ω with its center at $W_{n}^{i}$ and size of $2\times w_{m}$ and yields shortest distance to the current location $W_{n}^{i}$ is defined. The window Ω size is selected to ensure the safety distance to prevent the robot from colliding with the obstacles. The nearest narrow pixel $\left({\hat{x}}_{ns},{\hat{y}}_{ns}\right)$ corresponding with $W_{n}^{i}$ can be found by using Equation (7).

(7) $$({\hat{x}}_{ns},{\hat{y}}_{ns})=\underset{\begin{array}{l} (x_{ns}^{},y_{ns}^{})\in \Omega \\ Μ(x_{ns}^{},y_{ns}^{})=200 \end{array}}{\arg\min}(\sqrt{{\left(x_{n}^{i}-x_{ns}^{}\right)}^{2}+{\left(y_{n}^{i}-y_{ns}^{}\right)}^{2}},$$

(8) $$({\hat{x}}_{ns},{\hat{y}}_{ns})=\underset{\begin{array}{l} (x_{ns}^{},y_{ns}^{})\in \Omega \\ Μ(x_{ns}^{},y_{ns}^{})=100 \end{array}}{\arg\min}(\sqrt{{\left(x_{n}^{i}-x_{ns}^{}\right)}^{2}+{\left(y_{n}^{i}-y_{ns}^{}\right)}^{2}},$$

**Figure 14:**
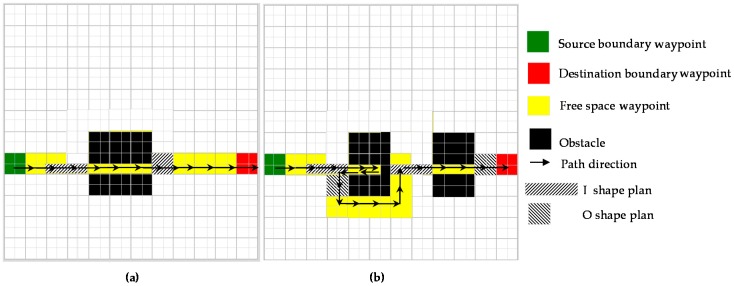
Covering narrow space constrains the proposed method. (**a**) Proposed method without obstacle from narrow space to waypoint, (**b**) proposed method with obstacle from narrow space to the waypoint.

Assuming that global path and shape planner is now sliding from left to right connecting $W_{n}(bl)$ and $W_{n}(br)$, the nearest narrow pixel $\left({\hat{x}}_{ns},{\hat{y}}_{ns}\right)$ within a window area Ω having the center at $W_{n}^{i}$ is already defined by the above algorithm. If there is no obstacle pixel in a range $\left(x_{n}^{i}:x_{n}^{br},y_{n}^{i}-w_{r}/2:y_{n}^{i}+w_{r}/2\right)$, the path and shape are planned as follows. Global shape planner will change the morphology plan of $W_{n}^{i}$ to the morphology having $w_{r}$ smaller than $v_{l}+v_{r}$. Then the global path planner will use A-star to traverse narrow space. When path $W_{n}^{i}$ reaches the last pixel point on the narrow space in front of boundary waypoint, the global shape planner is signaled that it is out of the narrow area. The shape plan at $W_{n}^{i}$ whose location offsets with $w_{r}$ to the right from the last narrow space is reshaped to the shape before entering the narrow area to keep the default morphology of hTetro. Then global path planner makes a path to connect the boundary waypoint as Figure 14a.

In the case of existing any obstacle pixel in range $\left(x_{n}^{i}:x_{n}^{br},y_{n}^{i}-w_{m}/2:y_{n}^{i}+w_{m}/2\right)$ as Figure 14b, the global planner will define waypoints based on the nearest obstacle within Ω with current global planner location $W_{n}^{i}$ as follows. The position of the nearest obstacle pixel $\left({\hat{x}}_{no},{\hat{y}}_{no}\right)$ can be found by Equation (8). After changing the shape at the position in front of the narrow space of distance $w_{m}$, the global planner will find the path by traversing the narrow area to the point $\left({\hat{x}}_{no}-w_{m}/2,{\hat{y}}_{no}\right)$ then returning to the point where the shape has changed and transform back to the default state. Since the path planning are based on the zigzag pattern, to simplify the zigzag pattern where each zigzag segment have the predefined width of O shape width, hTetro are transformed to default O shape after cover the narrow space. Moreover, transform from line I into a square O to avoid the collision during the pivot turn since square shape has the half-length comparing to the length of I shape. Then, to complete the trajectory from this point to boundary waypoint, the path plan will be calculated by using A-star as Figure 14b. The same mechanism can be applied for the case of moving from right to left.

Note that when moving into the narrow area, the proposed A-star based zigzag scanning algorithm described above can be applied to cover the entire space such as a scenario where a robot passes through narrow gate to new space.

## 5. Testbed Environment Setups

**Figure 15:**
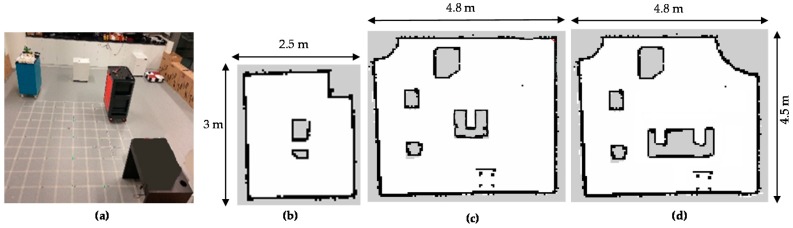
Testbed environment setups. (**a**) Real testbed environment, (**b**) small map with simple obstacles, (**c**) large map with moderate obstacles, (**d**) large maps with complicated obstacles.

To evaluate the preformation of the proposed global path and shape planner to cover the predefined areas, experimental environments are set up as Figure 15. The shape and size of the experimental environments are changed to create different maps. The small testbed map with simple shape obstacles has the size 4.5 m × 2.5 m. The large tested maps with moderate obstacles shapes and complicated obstacles shapes have the size 4.5 m × 4.8 m.

Static obstacles are placed arbitrarily in the testbed maps to create some narrow spaces. The scenarios with the dynamic obstacles are not taken in to account in this paper. The Hector mapping-based SLAM method is used to build maps for ROS navigation. The proposed method with the components of both the path plan and the shape plan was compared with the traditional A-star algorithm of ROS navigation and A-star based method [29]. The method A-star ROS navigation can be considered as heuristic approaches where A-star are used to find the shortest path to connect the left and right boundary waypoints staying on the same horizontal lines. In the future, more heuristic approaches will be taken in to account when considering the dynamic obstacles.

Waypoints are defined to create the roadmap by applying these methods before hTetro performs the navigation to clear the planned waypoints. During the hTetro navigation, the position of the robot after being finely tuned with AMCL was recorded to compute the area covered by the robot. To measure the covered areas, while the robot navigates, the path of the robot is widened to the size that corresponds to the actual width $w_{r}$ and marked in green color. Once the robot has completed the navigation of each test, the ratio $A_{r}$ between the covered areas and the need-to-be-covered areas and the ratio $R_{r}$ between the revisited areas and the covered areas will be calculated as Equations (9) and (10) respectively. Specifically, the areas covered by the robot during navigation are measured by the number of blue pixels (excluding blue pixels generated by the robot positions noise overlapped with obstacles, map borders and unidentified areas). The need-to-be-covered areas are the number of pixels corresponding to the free space areas with the value = 0 in the map on the ROS map server. The revisited areas are the overlapped areas of robot location during navigation. Since the percentage of the covered area showed differences between the tested methods, total path lengths can be evaluated through revisited areas. The higher the value of $A_{r}$, the better the tested method. The smaller $R_{r}$, the better the tested method. The processes are conducted ten times for each of these tested methods on each testbed map. The $A_{r}$ and $R_{r}$ ratios of all tested times are averaged to compare.

(9) $$A_{r}=\frac{Nunber\;of\;\mathrm{cov}ered\;pixels}{Nunber\;of\;free\;space\;pixels}\times 100\%$$

(10) $$R_{r}=\frac{Nunber\;of\;\mathrm{cov}ered\;pixels}{Nunber\;of\;free\;space\;pixels}\times 100\%$$

## 6. Experimental Results and Discussion

**Figure 16:**
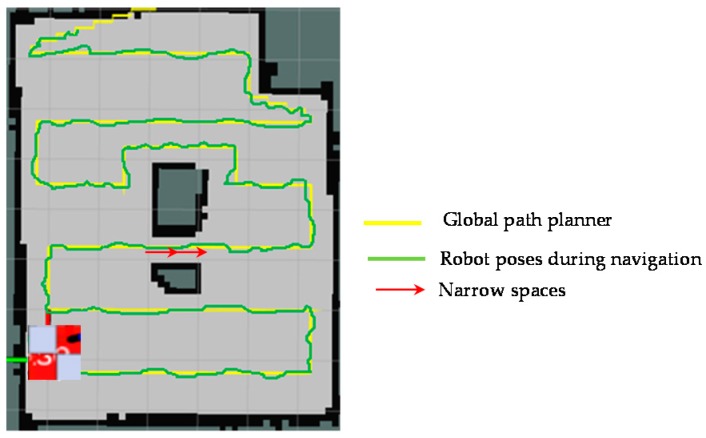
Global path planner and robot pose during navigation.

The results of forming the global path and shape plan of the proposed method are shown in Figure 16. The positions corresponding to boundary waypoints and narrow spaces are correctly defined. The planned trajectory is marked as yellow color; the narrow space areas are marked as red arrows. The green track in Figure 16 is the path that records the actual locations of the hTetro during the navigation to cover a map, given the path and shape planning. During the real-time implementation, the robot will follow the generated waypoints including both path and shape plans. Shape plan indicates the specific robot morphology at locations derived from the corresponding path plan. When the shape plan of the next waypoint indicates the change from one morphology to other, robot will perform the complete transformation to required morphology before navigating to next waypoint in path plan. It can be seen that hTetro with an onboard rpLidiar laser sensor and reconfiguration abilities can keep track and follow the global plan sufficiently with mirror errors.

**Figure 17:**
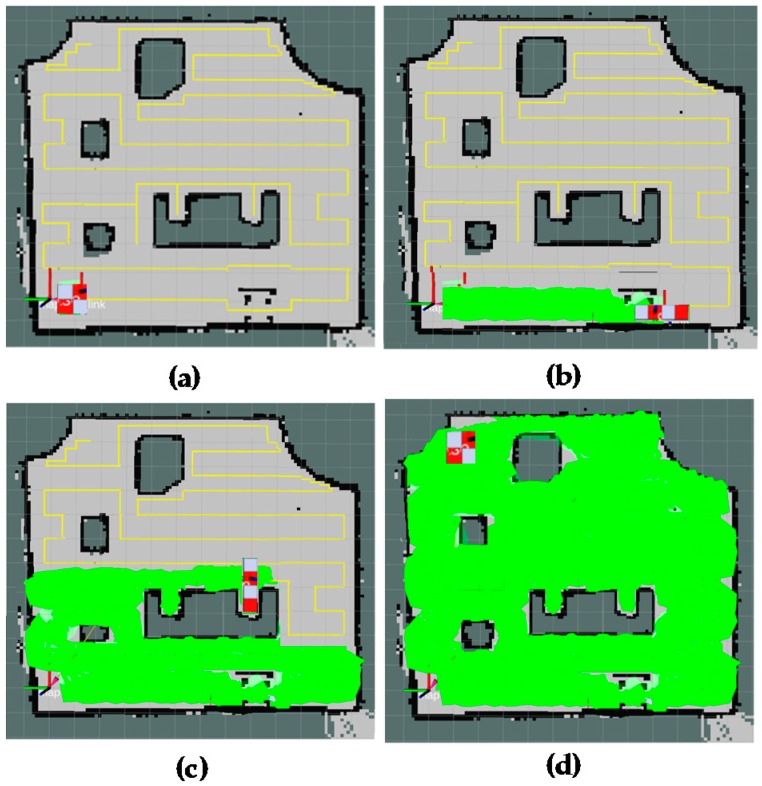
Areas coverage by the proposed method. (**a**) global path planning, (**b**) reconfiguration form O shape to horizontal I shape to cover narrow spaces, (**c**) reconfiguration form O shape to vertical I shape to cover narrow spaces, (**d**) total covered areas.

**Figure 18:**
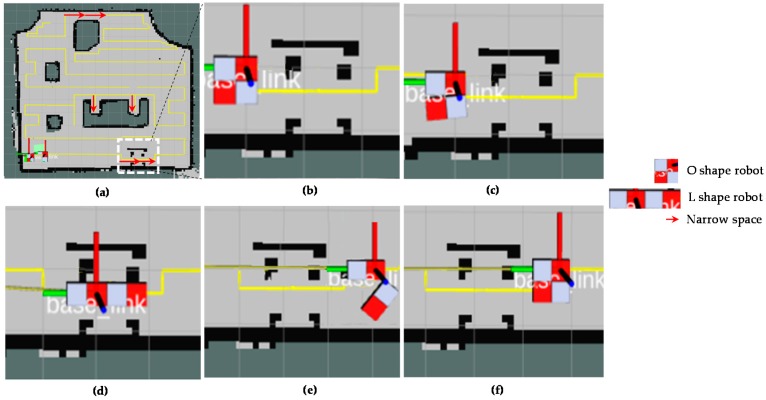
Morphology reconfigurations from O shape to horizontal I shape to navigate through the narrow spaces constraints on RVIZ. (**a**) Detected narrow spaces as red arrows, (**b**) the position before reconfiguring morphology, (**c**) starting morphology reconfiguration, (**d**) navigating with horizontal I shape, (**e**) Morphology reshapes to O after going out of narrow space, (**f**) keep navigating with O shape.

**Figure 19:**
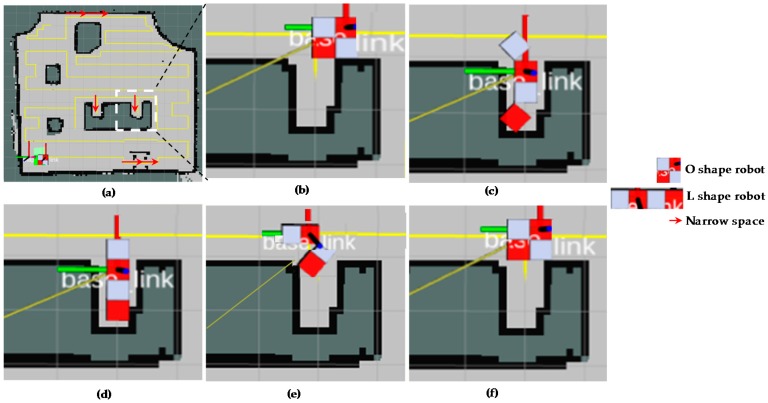
Morphology reconfigurations from O shape to vertical I shape to navigate through the narrow spaces constraints on RVIZ. (**a**) Detected narrow spaces as red arrows, (**b**) the position before reconfiguring morphology, (**c**) starting morphology reconfiguration, (**d**) navigating with vertical I shape, (**e**) Morphology reshapes to O after going out of narrow space, (**f**) keep navigating with O shape.

**Figure 20:**
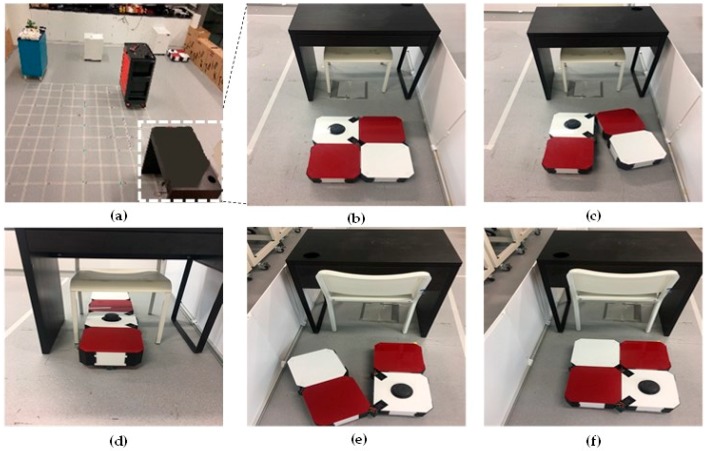
Morphology reconfigurations from O shape to vertical I shape to navigate through the narrow spaces constraints on real environment. (**a**) Detected narrow spaces as red arrows, (**b**) the position before reconfiguring morphology, (**c**) starting morphology reconfiguration, (**d**) navigating with vertical I shape, (**e**) Morphology reshapes to O after going out of narrow space, (**f**) keep navigating with O shape.

The Figure 17 provides visual views on how the robot follows the plan set by the global planner to cover the free space areas. From the Figure 17a–d, the free spaces including the narrow space areas are gradually covered by hTetro. The transformation steps of hTetro from O shape to horizontal I shape and from O shape to vertical I shape during autonomous navigation are provided on RVIZ a graphical monitoring tool of ROS in Figure 18 and Figure 19, respectively. The real pictures of hTetro reconfiguration steps to adapt with narrow spaces in the real environment can be observed in Figure 20. As per the results, the robot responds appropriately to morphology and path plan has been designed to cover almost all areas of narrow space. On the other hand, the robot cannot cover these spaces if it does not change its shape to suitable morphology.

**Figure 21:**
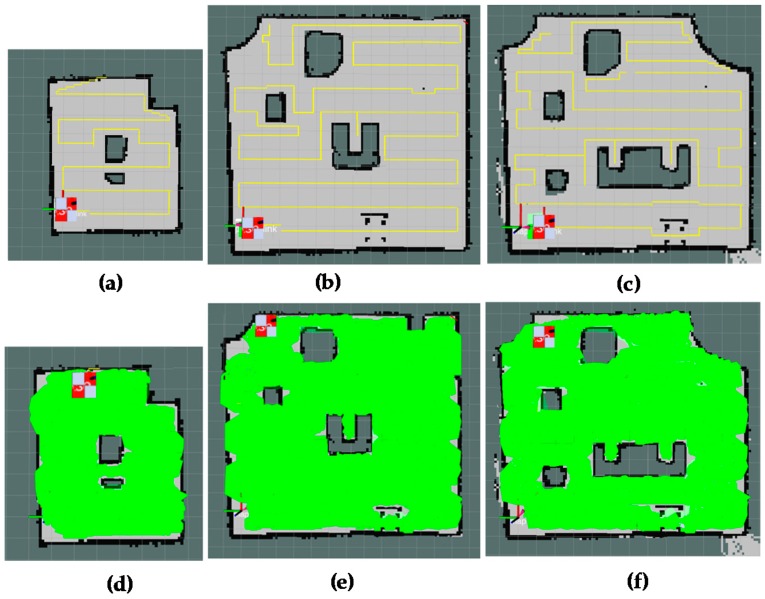
Areas coverage of the proposed method for different tested bed maps. (**a**,**d**) path planning and covered areas a small map with simple obstacles, (**b**,**e**) path planning and covered areas for a large map with moderate obstacles, (**c**,**f**) path planning and covered areas of for a large map with complicated obstacles.

The area coverage results of the proposed method for different map setups are presented in Figure 21. The results show the adaptive ability of the global planner with the diversity of settings from the small map with simple obstacle Figure 21a to moderate obstacle Figure 21b and complicated obstacle Figure 21c. Based on the generated plan, the hTetro can cover the free space areas almost completely.

**Figure 22:**
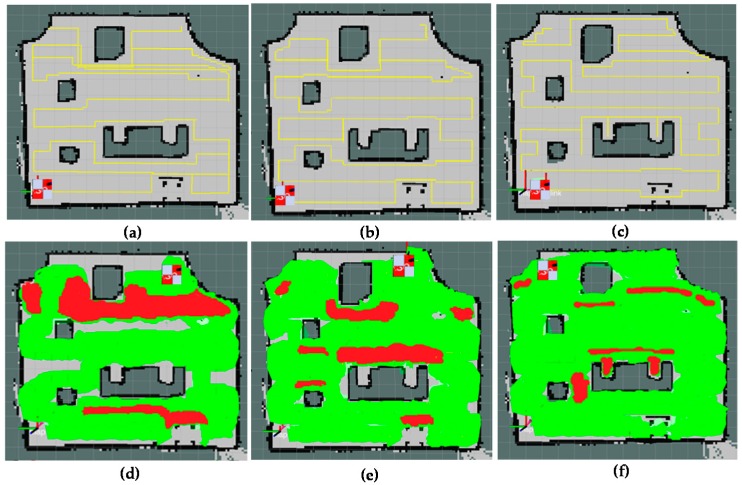
The area coverage comparisons. (**a**,**d**) path planning and covered areas of conventional A-star, (**b**,**e**) path planning and covered areas method [29], (**c**,**f**) path planning and covered areas of the proposed method with global path and shape planning.

The effectiveness of area coverage for tested methods is provided in Figure 22 for visual comparisons and in Table 1 and Table 2 for numerical comparisons. Covered areas by hTetro during navigation are marked as green color and the revisited areas which have been covered are marked as red color. Because of optimal the path planning enhanced by shape planning, the proposed method can achieve maximum area coverage and especially cover the area marked by red arrows in Figure 16. The ROS A-star based method and method in Reference [29] uncover some free space regions and revisit some covered areas. According to the Table 1, the coverage ratio of the proposed method archives the highest of 92.55%, while the method in Reference [29] without changing shape yield the second with a performance of 86.15% and the traditional A-star method only covers 72.63% of the total area and significantly lower than the proposed method.

Based on Table 2, the ratio of revisiting the area using the conventional A-star method degrades the robot performance by just achieving an average value of 21.72%. Since this method does not have any mechanism to avoid revisiting the covered cells while planning the shortest path by A-star. The segments of trajectory plan have a high probability of being overlapped in the cases of complicated obstacle shapes. While the method [29] which uses A-star for moving to the uncovered sub region have the similar issue of revisiting the covered cells, since the way-out from the last covered cell to first uncovered cell of another uncovered subarea requires the revisiting the covered cells. As per the results, the revisiting rate of this method was 12.26% and it is significantly higher than the proposed method. On the other hand, using modified A-star based zigzag scanning strategy to clear predefined the boundary waypoint and obstacle waypoints, the proposed method archives the lowest revisiting ratio of 5.25%.

The comparison of computing time to cover testbed environments with different space complexity between different algorithms are added in Table 3. Despite expensing extra time to complete the shapeshifting and to cover the narrow spaces, the computation time of proposed method yields just slightly higher than others do. The optimal path and shape plan for maximizing the area covering and reducing the revisited areas contributes to this result. Table 4 provides time consumption to generate the plan for different testbed maps. The proposed approach takes about 0.16 s on an average to generate the plan, which is feasible for real-time applications. Since we have validated the efficiency of the proposed method in three different scenarios, the average value in Table 1, Table 2, Table 3 and Table 4 was calculated for each tested method in order to normalize the performance metrics.

## 7. Conclusions

The novel proposed method of exploiting the Tetris inspired self-reconfigurable robot hTetro for optimizing area covering has proved its performance. The A-star zigzag scanning based global plan including path plan and shape plan showed the best results among the tested methods in terms of maximizing covered areas and reducing the revisiting of covered areas. The issues of covering the narrow space constrains are solved efficiently with the shape transformation of hTetro. The effectiveness of proposed method was demonstrated for the scenarios of static obstacles. The local path and shape planning to deal with both static and dynamic obstacles scenarios will be considered in the next researches.

## Figures and Tables

**Table 1:** The covered area ratio comparisons (%).

	A-Star ROSNavigation	Method [29]	Proposed Method with Path and Shape Planning
Small map with simple obstacles	80.12	90.23	95.32
Large map with moderate obstacles	72.21	85.68	93.12
Large map complicated obstacles	65.56	82.54	89.22
**Average**	**72.63**	**86.15**	**92.55**

**Table 2:** The revisited areas ratio comparisons (%).

	A-Star ROS Navigation	Method [29]	Proposed Method with Path and Shape Planning
Small map with simple obstacles	19.28	10.39	4.12
Large map with moderate obstacles	20.65	12.28	5.40
Large map complicated obstacles	25.24	14.12	6.22
**Average**	**21.72**	**12.26**	**5.25**

**Table 3:** The running comparisons of areas coverage (s).

	A-Star ROS Navigation	Method [29]	Proposed Method with Path and Shape Planning
Small map with simple obstacles	120.12	150.33	162.12
Large map with moderate obstacles	182.29	215.61	223.22
Large map complicated obstacles	215.56	262.54	269.16
**Average**	**172.66**	**209.50**	**218.17**

**Table 4:** The running comparisons of plan generating (s).

	A-Star ROS Navigation	Method [29]	Proposed Method with Path and Shape Planning
Small map with simple obstacles	0.11	0.13	0.15
Large map with moderate obstacles	0.13	0.15	0.16
Large map complicated obstacles	1.5	0.16	0.18
**Average**	**0.13**	**0.15**	**0.16**

## References

[1] Lumelsky V.J., Mukhopadhyay S., Sun K. (1990). Dynamic path planning in sensor-based terrain acquisition. IEEE Trans. Robot. Autom..

[2] Choset H., Pignon P. (1998). Coverage Path Planning: The Boustrophedon Cellular Decomposition. Field and Service Robotics.

[3] Acar E.U., Choset H., Rizzi A.A., Atkar P.N., Hull D. (2002). Morse Decompositions for Coverage Tasks. Int. J. Robot. Res..

[4] Oksanen T., Visala A. (2009). Coverage path planning algorithms for agricultural field machines. J. Field Robot..

[5] Choset H., Acar E., Rizzi A.A., Luntz J. Exact cellular decompositions in terms of critical points of Morse functions. Proceedings of the 2000 ICRA. Millennium Conference. IEEE International Conference on Robotics and Automation. Symposia Proceedings (Cat. No.00CH37065).

[6] Acar E.U., Choset H. Robust sensor-based coverage of unstructured environments. Proceedings of the 2001 IEEE/RSJ International Conference on Intelligent Robots and Systems. Expanding the Societal Role of Robotics in the the Next Millennium (Cat. No.01CH37180).

[7] Acar E.U., Choset H. (2002). Sensor-based Coverage of Unknown Environments: Incremental Construction of Morse Decompositions. Int. J. Robot. Res..

[8] Galceran E., Carreras M. Efficient seabed coverage path planning for ASVs and AUVs. Proceedings of the 2012 IEEE/RSJ International Conference on Intelligent Robots and Systems.

[9] Choset H., Burdick J. (2000). Sensor-Based Exploration: The Hierarchical Generalized Voronoi Graph. Int. J. Robot. Res..

[10] Wong S.C., MacDonald B.A. A topological coverage algorithm for mobile robots. Proceedings of the 2003 IEEE/RSJ International Conference on Intelligent Robots and Systems (IROS 2003) (Cat. No.03CH37453).

[11] Lee T.-K., Baek S.-H., Choi Y.-H., Oh S.-Y. (2011). Smooth coverage path planning and control of mobile robots based on high-resolution grid map representation. Robot. Auton. Syst..

[12] Oh J.S., Choi Y.H., Park J.B., Zheng Y.F. (2004). Complete coverage navigation of cleaning robots using triangular-cell-based map. IEEE Trans. Ind. Electron..

[13] Fazli P., Davoodi A., Pasquier P., Mackworth A.K. Complete and robust cooperative robot area coverage with limited range. Proceedings of the 2010 IEEE/RSJ International Conference on Intelligent Robots and Systems.

[14] Luo C., Yang S.X. A real-time cooperative sweeping strategy for multiple cleaning robots. Proceedings of the IEEE Internatinal Symposium on Intelligent Control.

[15] Sun Y., Ma S. ePaddle mechanism: Towards the development of a versatile amphibious locomotion mechanism. Proceedings of the 2011 IEEE/RSJ International Conference on Intelligent Robots and Systems.

[16] Nansai S., Rojas N., Elara M.R., Sosa R. Exploration of adaptive gait patterns with a reconfigurable linkage mechanism. Proceedings of the 2013 IEEE/RSJ International Conference on Intelligent Robots and Systems.

[17] Wei H., Cai Y., Li H., Li D., Wang T. Sambot: A self-assembly modular robot for swarm robot. Proceedings of the 2010 IEEE International Conference on Robotics and Automation.

[18] Kee V., Rojas N., Elara M.R., Sosa R. Hinged-Tetro: A self-reconfigurable module for nested reconfiguration. Proceedings of the 2014 IEEE/ASME International Conference on Advanced Intelligent Mechatronics.

[19] Prabakaran V., Elara M.R., Pathmakumar T., Nansai S. hTetro: A tetris inspired shape shifting floor cleaning robot. Proceedings of the 2017 IEEE International Conference on Robotics and Automation (ICRA).

[20] Prabakaran V., Elara M.R., Pathmakumar T., Nansai S. (2018). Floor cleaning robot with reconfigurable mechanism. Autom. Constr..

[21] Jelliss G.P. (1986). Chessics: The Journal of Generalised Chess, Special Issue on Chessboard Dis-sections.

[22] Duchoň F., Babinec A., Kajan M., Beňo P., Florek M., Fico T., Jurišica L. (2014). Path Planning with Modified a Star Algorithm for a Mobile Robot. Procedia Eng..

[23] Quigley M., Conley K., Gerkey B., Faust J. ROS: An open-source Robot Operating System. Proceedings of the Open-Source Software. Workshop of the International Conference on Robotics and Automation (ICRA).

[24] Grisettiyz G., Stachniss C., Burgard W. Improving Grid-based SLAM with Rao-Blackwellized Particle Filters by Adaptive Proposals and Selective Resampling. Proceedings of the 2005 IEEE International Conference on Robotics and Automation.

[25] Grisetti G., Stachniss C., Burgard W. (2007). Improved Techniques for Grid Mapping With Rao-Blackwellized Particle Filters. IEEE Trans. Robot..

[26] Fox D., Burgard W., Dellaert F., Thrun S. (1999). Monte Carlo Localization: Efficient Position Estimation for Mobile Robots. Proceedings of the Sixteenth National Conference on Artificial Intelligence and the Eleventh Innovative Applications of Artificial Intelligence Conference Innovative Applications of Artificial Intelligence.

[27] Kohlbrecher S., Meyer J., Graber T., Petersen K., Klingauf U., von Stryk O. (2013). Hector Open Source Modules for Autonomous Mapping and Navigation with Rescue Robots. RoboCup 2013: Robot World Cup XVII.

[28] Censi A. An ICP variant using a point-to-line metric. Proceedings of the 2008 IEEE International Conference on Robotics and Automation.

[29] Smith M., Baldwin I., Churchill W., Paul R., Newman P. (2009). The New College Vision and Laser Data Set. Int. J. Robot. Res..

[30] Viet H.H., Dang V.-H., Laskar M.N.U., Chung T. (2013). BA-star: An online complete coverage algorithm for cleaning robots. Appl. Intell..

